# A Prospective Interventional Study on the Beneficial Effect of Fish Oil-Enriched High-Protein Oral Nutritional Supplement (FOHP-ONS) on Malnourished Older Cancer Patients

**DOI:** 10.3390/nu17152433

**Published:** 2025-07-25

**Authors:** Hui-Fang Chiu, Shu Ru Zhuang, You-Cheng Shen, Subramanian Thangaleela, Chin-Kun Wang

**Affiliations:** 1 Department of Chinese Medicine, Taichung Hospital Ministry of Health and Welfare, Taichung 40301, Taiwan; huifangchiu@yahoo.com.tw; 2 School of Nutrition, Chung Shan Medical University, 110, Sec. 1, Jianguo North Road, Taichung 40201, Taiwanthangaleela@gm.csmu.edu.tw (S.T.); 3 School of Health Diet and Industry Management, Chung Shan Medical University, 110, Sec. 1, Jianguo North Road, Taichung 40201, Taiwan; youcheng@csmu.edu.tw

**Keywords:** cancer, medical nutrition therapy, fish oil, high protein, oral nutritional supplements, cancer-related fatigue

## Abstract

**Background:** Malnutrition and cancer-related fatigue (CRF) are prevalent in cancer patients, significantly impacting prognosis and quality of life. Oral nutritional supplements (ONSs) enriched with protein and ω-3 fatty acids may improve nutritional status and mitigate CRF. This study evaluates the effects of a high-protein, fish oil-enriched ONS (FOHP-ONS) on nutritional intake, body composition, fatigue, and quality of life in malnourished cancer patients. **Methods:** Cancer patients with malnutrition or inadequate food intake received 8 weeks of FOHP-ONS (2 cans/day, providing 4.2 g/day of ω-3 fatty acids). Dietary intake, body weight, handgrip strength, serum biochemical markers, nutritional status (PG-SGA), fatigue (BFI-T), and quality of life (EORTC QLQ-C30) were assessed at baseline, week 4, and week 8. **Results:** Of the 33 enrolled patients, 30 completed the study. Energy and protein intake significantly increased (*p* < 0.05), and body BMI and handgrip strength showed significant improvements (*p* < 0.05), while muscle mass did not change significantly. Nutritional status, assessed by PG-SGA, improved, with the proportion of severely malnourished patients (Stage C) decreasing from 46.7% to 13.3%, and moderately malnourished patients (Stage B) improving to well-nourished status (Stage A) from 10.0% to 30.0% (*p* < 0.001). Serum albumin levels increased significantly (*p* < 0.05), while fasting blood glucose significantly decreased (*p* < 0.05). Additionally, triglyceride levels significantly decreased (*p* < 0.05), while total cholesterol and LDL-C showed a downward trend. Cancer-related fatigue scores improved across all domains (*p* < 0.05), and quality of life significantly increased, particularly in physical and role functioning (*p* < 0.05). **Conclusions:** FOHP-ONS supplementation improved nutritional intake, body composition, and muscle strength while alleviating CRF and enhancing quality of life in malnourished cancer patients. These findings support its potential role in nutritional intervention for malnourished cancer patients.

## 1. Introduction

Cancer remains a major public health challenge, with an estimated 20 million new cases and 9.7 million cancer-related deaths worldwide in 2022 [1]. Malnutrition is a common and serious issue in cancer patients, affecting 20–70% depending on tumor type and disease stage [2]. Notably, 10–20% of cancer-related deaths are attributed to malnutrition rather than the disease itself [3]. Malnutrition in cancer patients is different from starvation-based malnutrition. Cancer-related malnutrition is different and has quite significant complications including anorexia, metabolic dysregulation, severe involuntary loss of skeletal muscle mass, loss of fat mass, systemic inflammation, and increased protein catabolism [4]. Another condition is multifactorial cachexia, which causes progressive functional loss, reduced quality of life, toxicity related to chemotherapy, reduced response to antineoplastic treatment, and reduced survival of patients [5]. Preventing or managing malnutrition is essential for improving patient outcomes. Medical nutrition therapy (MNT) is a widely recognized strategy for improving nutritional status in cancer patients. The European Society for Clinical Nutrition and Metabolism (ESPEN) guidelines recommend an energy intake of 25–30 kcal/kg BW/day and a protein intake of 1.0–1.5 g/kg BW/day for cancer patients. Fish oil supplementation has also been shown to stabilize or enhance appetite, food intake, lean body mass, and body weight [3,6]. However, many cancer patients struggle with inadequate food intake due to metabolic alterations, gastrointestinal dysfunction, treatment-related side effects, and hormonal imbalances [4,7]. ESPEN emphasizes the fortification of dietary intake, especially energy and protein, with a combination of ONSs and enteric tubes when patients unable to take supplements orally. ONS is an increasingly recognized strategy for treating malnutrition in patients to improve their nutrition intake and clinical status, and it is also cost-effective [8]. The importance of ONS in managing malnutrition among cancer patients is significant. Chronic inflammation associated with cancer complicates the nutritional status and makes the already available dietary methods insufficient. Malnourished patients require energy-dense, high-protein, ONS-enriched nutrients to overcome and combat the catabolic processes driven by cancer and its treatments. Therefore, the primary objective of this study is to evaluate whether energy-dense, high-protein oral nutritional supplements (ONSs) enriched with fish oil can improve nutritional status, food intake, body weight, and related body composition parameters in malnourished cancer patients.

A systemic review and meta-analysis study in 2018 evaluated the impact of ONS on body weight in cancer patients undergoing chemotherapy. Randomized control trial studies focused on high-energy ONSs, with high-protein ONSs enriched with n-3 polyunsaturated fatty acids (PUFAs) taken for analysis. Their findings showed significant increase in body weight among patients receiving high-protein, PUFA-enriched ONSs [9]. Another dose–response meta-analysis of randomized clinical trials carried out by Habibi et al. [10] found that ONSs significantly improved weight gain, fatigue scores, PG-SGA scores, and QOL. Dosage of ≥400 mL per day significantly reduced fatigue, and ≥500 mL per day improved QOL score significantly.

Cancer-related fatigue (CRF) is another critical but often overlooked complication. It is a persistent, distressing fatigue that significantly impairs daily functioning and quality of life [11]. CRF affects 25–99% of patients, with severe cases persisting for months or even years after treatment [12,13,14]. Despite its high prevalence, effective treatment options for CRF remain limited [15]. Pharmacologic interventions provide minimal benefits and may pose risks of toxicity or drug interactions, while psychological interventions and exercise require high adherence [15,16]. Treatment for CRF is often aimed at reducing inflammation. Dietary patterns such as a Mediterranean diet and other plant-based diets appear to reduce CRF [15]. Cancer patients and cancer survivors need more protein due to their abnormal protein metabolism, increased protein turnover, as well as gluconeogenesis of amino acids [17]. Increased protein intake supports preservation of lean mass and body composition. Protein intake helps to maintain lean mass and reduce CRF [18]. However, the effects of a high-protein, ω-3 fatty acids-enriched ONS on CRF in cancer patients remain unclear. Thus, the secondary objective of this study is to assess whether such supplementation improves CRF and quality of life in cancer patients.

## 2. Materials and Methods

### 2.1. Human Trial Study

This is a prospective, single-arm, multicentered study. Participants were recruited from Chung Shan Medical University’s Department of Radiation Oncology and nursing home facilities in Taichung and Changhua, Taiwan. Inclusion criteria included patients aged 20–80 years who had been diagnosed with any type of cancer and exhibited issues such as underweight, poor nutritional status, or insufficient food intake. Exclusion criteria included pregnancy or lactation, immune system disorders, severe diabetes (HbA1c ≥ 9%), insulin-dependent diabetes mellitus, or supplementation with ω-3 fatty acid, arginine, or glutamine within one month prior to intervention. The study was approved by the Institutional Review Board of Chung Shan Medical University Hospital (IRB Approval number: CS1-21130). The study subjects were recruited and before the trial, subjects were informed of the details of the trial, their cooperation, implementation methods, and their rights. After confirmation, the subjects signed the informed consent and provided their basic information.

### 2.2. Intervention

During 8 weeks of intervention, participants received 2 cans/day of high-protein and energy-dense oral nutritional supplements containing re-esterified triglyceride fish oil (FOHP-ONS), providing a total of 4.2 g/d ω-3 (Table 1) (LivStrong Cancer Liquid Formula, Nutritec-Enjoy Corporation, Taipei, Taiwan). Patients were asked not to consume any other nutritional supplements or functional food during the study period. Registered dietitians provided dietary counseling to all participants to ensure their overall dietary intake, including 2 cans of FOHP-ONS, adhered to the ESPEN guidelines for cancer patients [4,6]. Compliance with the intervention was closely monitored through weekly phone calls. Additionally, any adverse events or gastrointestinal side effects associated with FOHP-ONS were recorded daily by caregivers. Pre-existing gastrointestinal symptoms due to prior treatments or underlying conditions were documented, and only symptom exacerbation following FOHP-ONS was classified as an adverse event. Gastrointestinal symptoms were defined as follows: diarrhea (≥200 g/day of loose stools), bloating (abdominal distension due to gas or fluid), constipation (<3 bowel movements/week with straining or hard stools), nausea, vomiting, and indigestion (upper abdominal discomfort, bloating, or pain).

### 2.3. Measurement

Participants were evaluated at three time points: baseline, midpoint (Week 4), and the end of the intervention (Week 8). Anthropometric measurements, including body weight, height, body mass index (BMI), mid-arm circumference (MAC), mid-arm muscle circumference (MAMC), and triceps skinfold thickness (TSF) were calculated following standardized protocols. After obtaining the height and weight measurement, their BMI (weight/height squared, kg/m^2^) was calculated. TSF was measured using a skinfold caliper, averaging the two closest values from three readings. MAC was measured at the upper arm midpoint, and MAMC was calculated as MAMC (cm) = MAC (cm) − (0.314 × TSF (cm)); the triceps skinfold thickness (TSF) was tested by the measurer using the thumb and index finger of the left hand to vertically pinch the skin layer about 1 cm above the middle of the right arm of the subject, making sure to only pull up the fat layer. Then the measurer pressed the spring clip with their right hand and, after pinching the skin layer, released their thumb and read the scale indicated by the pointer after 2 s. After which they pressed the spring clip and removed the measuring device. The measuring process was repeated three times in a row and the two most recent values were averaged. Muscle strength was assessed using a handgrip dynamometer (Grip-D, TKK 5101, Takei Scientific Instruments Co., Ltd, Shinagawa-ku, Tokyo, Japan). Mercury blood pressure gauge was to measure the systolic and diastolic pressure of the right arm. Before measurement, the measurer made sure that the cuff was completely deflated.

Biochemical analyses were performed on blood samples collected in the morning after an overnight fast. These included assessments of serum protein levels, high-sensitivity C-reactive protein (hs-CRP), fasting blood glucose, glycated hemoglobin (HbA1c), insulin levels, and insulin resistance, which was calculated using the homeostasis model assessment of insulin resistance (HOMA-IR). HOMA-IR was calculated by using the formula fasting insulin (μU/mL) × fasting blood glucose (mmol/L)/22.5 g. Lipid profiles included triglycerides, total cholesterol, high-density lipoprotein cholesterol (HDL-C), and low-density lipoprotein cholesterol (LDL-C).

Nutritional status was assessed by using the Scored Patient-Generated Subjective Global Assessment (PG-SGA). This questionnaire consists of two parts. The first part was answered by the patient, providing past weight changes, food intake, symptoms affecting food intake, and changes in physical functions. The rest was completed by oncologists, nurses, and dietitians. After obtaining the overall score, the patients were evaluated into 3 stages. Stage A (0–3 points) represents well-nourished; Stage B (4–8 points) represents moderately malnourished or at risk of malnutrition; Stage C (≥9 points) represents severely malnourished. A core questionnaire for quality of life with 30 core questions (QLQ-C30) developed by the European Organization for Research and Treatment of Cancer (EORTC) was used evaluate multiple dimensions of QOL [19]. The QLQ-C30 consists of 30 items divided into global health status/QOL, 5 functional scales (physical, role, emotional, cognitive and social functioning), 3 symptom scales (fatigue, pain and nausea/vomiting), and 6 single-item measures (dyspnea, insomnia, appetite loss, constipation, diarrhea, and financial difficulties). Each scale and single-item measure is scored from 0 to 100, with higher scores reflecting a higher level of functioning or severity of symptoms.

**Figure 1:**
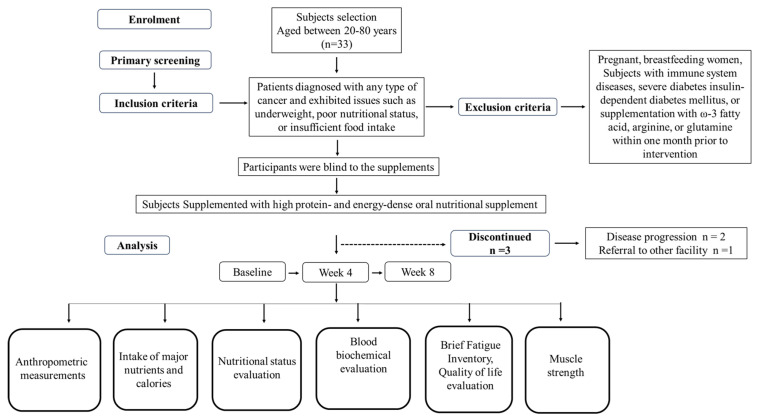
The illustration shows the study protocol.

Cancer-related fatigue was assessed using the Taiwan version of the Brief Fatigue Inventory (BFI-T), a validated instrument designed to evaluate fatigue severity and its interference with daily life over the preceding 24 h [20]. The BFI-T consists of two sections: fatigue severity and fatigue interference. The fatigue severity section includes three items that measure the following: (1) fatigue right now, (2) usual fatigue in the last 24 h, and (3) the worst fatigue in the last 24 h. Each item is scored on a 0 to 10 scale, where 0 represents “no fatigue” and 10 indicates “fatigue as bad as you can imagine”. The fatigue interference section evaluates the impact of fatigue on six aspects of daily life: (1) general activities, (2) mood, (3) walking ability, (4) normal work (including both work outside the home and daily chores), (5) relations with other people, (6) and enjoyment of life. Each interference item is scored on a scale from 0 to 10, with higher scores reflecting greater interference. The total fatigue score is calculated as the average of all items, with fatigue classified into three categories based on the total score: mild (1–3), moderate (4–6), and severe (7–10). The complete work plan is detailed in Figure 1.

### 2.4. Statistical Analysis

Statistical analyses were performed using the Statistical Package for the Social Sciences (SPSS, version 18.0 for Windows; SPSS Inc, Chicago, IL, USA). Data normality was assessed using the Shapiro–Wilk test prior to analysis. For repeated measures with a normal distribution, differences across time points (baseline, midpoint, and endpoint) were analyzed using one-way repeated measures ANOVA. For non-normally distributed data, Friedman’s test was applied to evaluate differences across the 3 time points. *p* values < 0.05 were considered statistically significant. Results are expressed as mean ± standard deviation (Mean ± SD). Descriptive statistics were used for the analysis of non-quantitative variables.

## 3. Results

A total of 33 patients were initially enrolled in this study. During the trial, 3 patients withdrew due to disease progression (*n* = 2) or referral to other facility (*n* = 1). Consequently, 30 patients completed the study and were included in the final analysis (Figure 1). The study cohort consisted of 21 men and 9 women, with a mean age of 67.90 ± 8.86 years (range from 47 to 80 years). At baseline, 43.3% of patients were categorized as moderately malnourished or at risk of malnutrition, while 46.7% were classified as severely malnourished based on PG-SGA. Additionally, patients mean BMI was 19.98 ± 2.96, while 33.3% were underweight (BMI < 18.5 kg/m^2^) (Table 2).

### 3.1. Compliance and Tolerance

Compliance with FOHP-ONS was assessed weekly through telephone follow-ups, with a compliance rate of 99.8 ± 0.50% throughout the intervention. No gastrointestinal side effects, including nausea, vomiting, constipation, diarrhea, bloating, or abdominal pain, were reported as being caused by FOHP-ONS during the study period.

### 3.2. Nutritional Intake and Energy Balance

At baseline, patients had significantly lower energy (1458.03 ± 138.65 kcal/day) and protein intake (60.81 ± 10.80 g/day) than ESPEN-recommended targets (1716.93 ± 160.44 kcal/day and 86.00 ± 8.46 g/day) (*p* < 0.05), with 40.0% and 83.3% failing to meet caloric and protein needs (Table 2 and Table 3). After 8 weeks of intervention, energy and protein intake significantly increased to 1713.58 ± 132.27 kcal/day and 86.23 ± 5.70 g/day (*p* < 0.05). Adjusted for ideal body weight (IBW), energy intake increased from 25.64 ± 2.92 to 29.99 ± 0.84 kcal/kg IBW/day, and protein intake improved from 1.06 ± 0.18 to 1.50 ± 0.07 g/kg IBW/day (*p* < 0.05). Fat intake also showed a significant increase (45.50 ± 9.83 to 61.82 ± 4.60 g/day, (*p* < 0.05), while carbohydrate intake exhibited a modest rise (Table 3).

### 3.3. Nutritional Status

**Figure 2:**
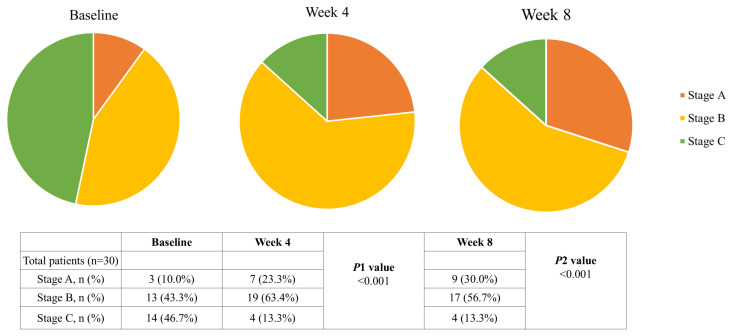
The stages of nutritional status evaluated by PG-SGA are described as a pie chart. *p*1 value indicates the comparison between baseline and week 4 (Chi-square test). *p*2 value indicates the comparison between baseline and week 8 (Chi-square test).

At baseline, mean BMI was 19.98 ± 2.96 kg/m^2^, with 33.3% classified as underweight. After 8 weeks, body weight and BMI significantly increased to 52.29 ± 8.08 kg and 20.17 ± 2.97 kg/m^2^, respectively (*p* < 0.05). Similarly, BMI increased from 19.98 ± 2.96 kg/m^2^ at baseline to 20.06 ± 2.95 kg/m^2^ at week 4 and 20.17 ± 2.97 kg/m^2^ at week 8 (*p* < 0.05). Muscle mass (MAMC) also improved from 20.43 ± 2.82 to 20.57 ± 2.74 cm, while grip strength increased from 14.08 ± 8.39 kg to 15.98 ± 9.56 kg (*p* < 0.05) (Table 4). Nutritional status, assessed by PG-SGA, improved significantly. The proportion of well-nourished patients (Stage A) increased from 10.0% at baseline to 30.0% at week 8, while those classified as severely malnourished (Stage C) declined from 46.7% to 13.3% (*p* < 0.001) (Figure 2). Nonetheless, Serum albumin levels, commonly used to evaluate long-term protein status, increased significantly throughout the intervention. At baseline, 14 participants (46.7%) exhibited serum albumin levels below the normal range. By week 8, mean albumin levels increased from 3.51 ± 0.44 g/dL to 3.66 ± 0.35 g/dL (Table 4).

### 3.4. Biochemical Parameters

After 8 weeks of FOHP-ONS supplementation, hs-CRP levels showed a decreasing trend (0.86 ± 1.43 to 0.71 ± 1.02 mg/dL), though not statistically significant. Fasting blood sugar significantly decreased from 95.70 ± 16.38 mg/dL at baseline to 91.40 ± 13.12 mg/dL at week 8 (*p* < 0.05). Although HbA1c and HOMA-IR index showed a trend of reduction, the changes were not statistically significant. Regarding lipid profiles, triglyceride levels significantly decreased from 99.63 ± 44.00 mg/dL at baseline to 84.23 ± 33.82 mg/dL at week 8 (*p* < 0.05). Total cholesterol and LDL-c levels also exhibited a downward trend, while HDL-c levels remained stable throughout the intervention (Table 4).

### 3.5. Cancer-Related Fatigue

For fatigue severity, “fatigue right now” decreased from 5.93 ± 1.96 at baseline to 5.07 ± 1.68 at week 8 (*p* < 0.05). Similarly, “usual fatigue in the last 24 h” improved from 5.23 ± 2.46 at baseline to 4.43 ± 1.85 at week 8 (*p* < 0.05), and “the worst fatigue in the last 24 h” declined from 6.87 ± 1.78 to 6.07 ± 1.74 (*p* < 0.05). Functional interference also decreased across all domains, including general activities, mood, walking, normal work, relations with other people, and enjoyment of life (*p* < 0.05 for all). The overall interference score declined from 5.07 ± 2.01 to 4.04 ± 1.76 (*p* < 0.05) (Table 5).

### 3.6. Quality of Life

The global health status/QoL score increased from 44.72 ± 14.60 at baseline to 49.72 ± 12.66 at week 4 and further to 51.94 ± 12.12 at week 8 (*p* < 0.05). Among the functional scales, physical functioning improved significantly from 25.78 ± 22.74 at baseline to 38.00 ± 19.11 at week 4 and 50.44 ± 20.39 at week 8 (*p* < 0.05). Similarly, role functioning increased from 27.22 ± 28.19 to 32.78 ± 26.07 at week 4 and 47.22 ± 30.98 at week 8 (*p* < 0.05). Cognitive, emotional, and social functioning also demonstrated notable improvements, with cognitive functioning rising from 56.11 ± 21.66 at baseline to 76.11 ± 26.87 at week 8 (*p* < 0.05) and emotional functioning increasing from 60.00 ± 19.38 to 81.11 ± 24.07 at week 8 (*p* < 0.05). On the symptom scales, significant reductions were observed in constipation, which decreased from 32.22 ± 16.34 at baseline to 16.67 ± 19.08 at week 4 and 13.33 ± 18.77 at week 8 (*p* < 0.05). Diarrhea and dyspnea scores also improved, decreasing from 17.78 ± 16.91 and 28.89 ± 25.87 at baseline to 15.56 ± 19.04 and 16.67 ± 25.89 at week 8, respectively (*p* < 0.05 for both). Fatigue showed substantial improvement, decreasing from 65.93 ± 17.25 at baseline to 45.56 ± 20.91 at week 4 and 34.81 ± 19.29 at week 8 (*p* < 0.05). Other symptoms, including pain, insomnia, appetite loss, and financial difficulties, also exhibited improvements (Table 6).

## 4. Discussion

Studying the nutritional status of cancer patients before and during the treatment is an important strategy in cancer care, as therapeutic efficacy is greatly influenced by nutritional status. ONSs fortified with ω-3 fatty acids showed anti-inflammatory functions. A cereal-based ONS containing chia seed powder rich in ω-3 fatty acids was found to lower pro-inflammatory cytokines in lipopolysaccharide-stimulated peripheral blood mononuclear cells (PBMCs) isolated from cancer patients [21].

In this study, we demonstrated an 8-week intervention with a high-protein and energy-dense oral nutritional supplement containing re-esterified triglyceride fish oil (FOHP-ONS), which significantly improved nutritional intake, body composition, biochemical parameters, cancer-related fatigue, and quality of life in malnourished cancer patients. Fatigue severity and its impact on daily activities were notably reduced, while multiple functional domains of quality of life showed significant improvement. To our knowledge, this is the first clinical study to demonstrate the benefits of a high-protein, energy-dense ONS enriched with ω-3 fatty acid in alleviating CRF and enhancing quality of life in cancer patients. These findings highlight the potential role of FOHP-ONS as a nutritional strategy to support cancer patients’ overall well-being.

Malnutrition in cancer patients is associated with poor treatment tolerance, increased infection and complications, prolonged hospitalization, and higher mortality [3,22,23,24]. In this study, 8 weeks of FOHP-ONS supplementation significantly improved energy and protein intake, leading to increased body weight, BMI, and muscle mass. Serum albumin, a key marker of nutritional status, also increased. Previous studies have shown that lower albumin levels are associated with increased inflammation, poor prognosis, and higher mortality risk in cancer patients [22,25,26]. The improvement in albumin levels, along with enhanced nutritional intake, suggests a positive impact on overall nutritional status. On the one hand, albumin and prealbumin were considered as more frequent markers for accessing nutritional status of cancer patients. The blood albumin levels were found to be influenced by various physiological factors like age, circulating estrogen, loss of blood, as well as iron deficiency [27]. Thus, on the other hand, the decision to include albumin as an important serum marker for nutritional status in cancer patients cannot be an accurate one. There are other studies that also studied albumin concentration with nutritional and inflammatory markers to evaluate the ONS intervention [28,29]. A meta-analysis study in older adults with cancer showed the nutritional index was based on albumin and lymphocyte counts [30]. Additionally, PG-SGA scores demonstrated a significant reduction in severe malnutrition, with more patients shifting toward moderate malnutrition (Stage B) or achieving well-nourished status (Stage A). These findings highlight the role of FOHP-ONS in supporting nutritional status in malnourished cancer patients.

Muscle loss and decreased strength affect 20–70% of cancer patients, contributing to fatigue, impaired function, reduced treatment tolerance, and increased mortality [3,22,25,31]. While cancer was once thought to completely suppress muscle protein synthesis, research shows it remains possible despite aging, inactivity, systemic inflammation, and insulin resistance. However, as disease severity progresses, the anabolic threshold rises, making muscle maintenance more difficult [3,32,33]. Adequate energy and protein intake are essential for muscle synthesis and improving strength and mass [32,34]. Protein quality also plays a crucial role, with the protein digestibility-corrected amino acid score (PDCAAS) indicating its ability to support nitrogen balance (1 = highest quality). High-quality proteins enable patients to meet essential amino acid (EAA) requirements with lower intake, which reduces dietary burden. Previous studies have shown that 14 g of EAAs enhances whole-body protein synthesis and net anabolism in advanced lung cancer patients [35]. Among EAAs, leucine is a key regulator of mTORC1, which promotes muscle protein synthesis while inhibiting catabolism [36]. Clinical studies have demonstrated that leucine supplementation improves body weight, BMI, fat-free mass, and skeletal muscle mass in gastrointestinal cancer patients undergoing chemotherapy [37]. In this study, FOHP-ONS contains casein, a high-quality protein source (PDCAAS = 1) [38], providing EAAs (3835 mg/100 mL, 18,178 mg/day) and leucine (861 mg/100 mL, 4081 mg/day). Over a 12-week intervention, muscle mass showed a modest increase, while handgrip strength improved significantly. These findings suggest that FOHP-ONS may help preserve muscle mass and enhance strength in malnourished cancer patients.

The health benefits of ω-3 fatty acids in cancer patients have been well elucidated, including their ability to reduce inflammation, inhibit tumor growth, decrease protein and fat catabolism, and enhance muscle protein synthesis [39,40]. According to the latest ESPEN guidelines, ω-3 supplementation in advanced cancer patients undergoing chemotherapy, particularly those at risk of weight loss or malnutrition, may help stabilize or improve appetite, food intake, lean body mass, and body weight [3]. Furthermore, multiple studies have demonstrated that ω-3 fatty acids can alleviate cancer cachexia, neuropathic toxicity, and postoperative complications while enhancing overall survival and quality of life [6,41,42,43]. Although the optimal dosage remains uncertain, the 2017 ESPEN guidelines recommend 1–2 g/day to mitigate inflammation, while higher doses exceeding 2 g/day are required for clinically significant nutritional benefits [6]. In this study, participants received 4.2 g/day of ω-3 fatty acids in accordance with ESPEN recommendations. The results align with the previous findings demonstrating improvements in body weight, muscle mass, and appetite. These benefits contribute to enhanced quality of life and reduction in CRF.

Unexpectedly, our study found a significant reduction in triglyceride levels among participants. Although changes in total cholesterol, LDL-C, and HDL-C were not statistically significant, total cholesterol and LDL-C showed a decreasing trend, whereas HDL-C showed an increasing trend. These findings may be attributed to the effects of ω-3 fatty acids, which are well documented for their triglyceride-lowering properties. The mechanisms underlying this effect include inhibition of hepatic lipogenesis and VLDL production, enhanced TRL lipolysis, increased anti-inflammatory lipid mediators, and reduced thrombosis [44]. The lipid-modulating effects of ω-3 fatty acid supplementation have been demonstrated across various populations, including healthy individuals, patients with dyslipidemia, and those with diabetes [44,45,46,47]. However, clinical studies on the lipid-lowering effects of ω-3 fatty acid in cancer patients remain limited. Hyperlipidemia is a common comorbidity among cancer patients and survivors. Even in those without pre-existing dyslipidemia, tumor progression and various anticancer drugs have been associated with lipid abnormalities. Emerging evidence suggests that hyperlipidemia may contribute to poor cancer outcomes by promoting tumor invasion and metastasis, reducing treatment efficacy, and increasing cardiovascular toxicity [48,49]. Given its favorable safety profile, ω-3 fatty acid supplementation may be a potential strategy for managing dyslipidemia in cancer patients.

Additionally, our study demonstrated a significant reduction in fasting blood glucose following 12 weeks of FOHP-ONS supplementation, along with decreases in insulin levels and HOMA-IR index. These improvements may be linked to a lower proportion of daily energy intake from carbohydrates and a higher proportion from fats. The ESPEN guidelines recommend adjusting macronutrient composition in weight-losing cancer patients with insulin resistance by increasing fat intake while reducing carbohydrate intake to enhance energy density and lower the glycemic load [3]. Similarly, the ESMO guidelines suggest that fat-rich diets allow for smaller meal volumes while maintaining adequate caloric intake, recommending that fat contributes half of the non-protein caloric intake in patients with cachexia [25]. The intervention in this study provided 39.5% of total energy from fat and 35% from carbohydrates, aligning with these recommendations. This macronutrient distribution likely contributed to improved glycemic control by reducing carbohydrate energy distribution and minimizing glycemic fluctuations [50].

CRF severity is associated with poorer quality of life [51] and linked to malnutrition and inflammation, as reflected in higher PG-SGA scores [52], hypoalbuminemia, and elevated CRP levels [53]. Optimizing nutritional intake and modulating inflammation may be effective strategies for CRF management. In our study, FOHP-ONS supplementation significantly improved nutritional status, which may have contributed to reductions in CRF and enhancements in quality of life. Another possible factor is ω-3 fatty acid supplementation. Several studies have demonstrated its potential to alleviate CRF and improve quality of life [54,55,56]. A consensus statement from the Taiwan Society of Cancer Palliative Medicine and the Taiwan Oncology Nursing Society also supports the use of ω-3 fatty acids for CRF management (level of evidence IB; grade of recommendation B) [57]. These findings highlight the potential of ω-3 fatty acids as part of a comprehensive nutritional intervention to mitigate CRF and improve overall well-being in cancer patients.

This study has some limitations. First, the single-arm design without a control group limits the ability to establish a direct causal relationship between FOHP-ONS and the observed outcomes Therefore, larger randomized controlled trials are recommended to validate these findings further to elucidate the specific mechanisms of FOHP-ONS. Second, the intervention period was only eight weeks, which may not have been sufficient to assess long-term effects on the outcomes. Lastly, our study did not restrict enrollment based on cancer type or stage, which may have introduced variability into the results. Future research should account for these factors to enhance the reliability and applicability of findings.

## 5. Conclusions

The findings from this study demonstrated that an 8-week intervention with FOHP-ONS, a high-protein, energy-dense oral nutritional supplement enriched with ω-3 fatty acids, may offer multiple clinical benefits in malnourished cancer patients. Significant improvements were observed in dietary intake and protein intake as well as in BMI and handgrip strength, indicating positive effects. The proportion of stage C patients with severe malnutrition was reduced substantially, and well-nourished stage A individuals increased. This supports the role of supplements in improving overall nutrition status. Biochemically, significant increases in serum albumin and reductions in triglyceride levels and fasting blood glucose were influenced by ω-3 fatty acids. Furthermore, inclusion of ω-3 fatty acids was associated with improvements in appetite and quality of life, as well as a possible reduction in CRF. These findings suggest that FOHP-ONS may serve as a comprehensive nutritional intervention to improve overall well-being in cancer patients.

## Figures and Tables

**Table 1:** Composition of high-protein oral nutritional supplementation.

Formula Description	FOHP-ONS (237 mL/can)
Energy (kcal/100 mL)	150
Energy-dense (kcal/cc)	1.5
Macronutrients	
Total protein (g/100 mL)	9.5
Total carbohydrate (g/100 mL)	13.7
Dietary fiber (g/100 mL)	1.1
Total lipid (g/100 mL)	6.6
ω-3 fatty acid (g/100 mL)	0.9
EPA (g/100 mL)	0.5
DHA (g/100 mL)	0.1

Kcal—kilocalories; cc—cubic centimeter; EPA—eicosapentaenoic acid; DHA—docosahexaenoic acid.

**Table 2:** Baseline characteristics.

Description	Intervention Group (*n* = 30)
Age, y	67.90 ± 8.86
Female, *n* (%)	9 (30%)
Current Treatment, *n*	
Chemotherapy	7
Surgery	9
Radiotherapy	2
Surgery + radiotherapy	6
Surgery + radiotherapy + chemotherapy	1
None	5
Nutritional Status	
BMI (kg/m^2^)	19.98 ± 2.96
BMI < 18.5, *n* (%)	10 (33.3%)
Albumin (g/dl)	3.51 ± 0.44
PG-SGA, *n* (%)	
Stage A (well-nourished)	3 (10.0%)
Stage B (moderate/suspected malnutrition)	13 (43.3%)
Stage C (severely malnourished)	14 (46.6%)
Insufficient Food Intake, *n* (%)	
Energy intake < 25 kcal/BW kg/d	12 (40.0%)
Protein intake < 1.2 g/BW kg/d	25 (83.3%)

Data are presented in numbers and proportions (%) and as mean ± standard deviation. BMI—body mass index.

**Table 3:** Daily energy and macronutrients intake.

	Intervention Group (*n* = 30)	
Energy goal (30 kcal/kg BW/d) ^#^	1716.93 ± 160.44	
Protein goal (1.2 g/kg BW/d) ^#^	86.00 ± 8.46	
Actual intake	Baseline	Week 8
Energy (kcal)	1458.03 ± 138.65 *^b^	1713.58 ± 132.27 ^a^
Energy (kcal/kg IBW)	25.64 ± 2.92	29.99 ± 0.84
Protein (g/d)	60.81 ± 10.80 *^b^	86.23 ± 5.70 ^a^
Protein (g/kg IBW)	1.06 ± 0.18	1.50 ± 0.07
Carbohydrate (g/d)	193.69 ± 22.10	199.82 ± 21.74
Fat (g/d)	45.50 ± 9.83 ^b^	61.82 ± 4.60 ^a^

Data are presented as mean ± standard deviation. ^#^ Goals are based on ESPEN cancer guidelines [3]. * *p* value indicates a significant difference (*p* < 0.05) from recommended goals (paired *t*-test). Different superscript letters in the group indicate statistically significant differences between baseline and week 8. BW, body weight; IBW, ideal body weight.

**Table 4:** Body composition and biochemical parameters.

	Baseline	Week 4	Week 8
Body Composition			
Body weight (kg)	51.81 ± 8.28 ^c^	52.04 ± 8.23 ^b^	52.29 ± 8.08 ^a^
BMI (kg/m^2^)	19.98 ± 2.96 ^c^	20.06 ± 2.95 ^b^	20.17 ± 2.97 ^a^
MAC (cm)	24.59 ± 3.34 ^b^	24.64 ± 3.34 ^b^	24.80 ± 3.18 ^a^
MAMC (cm)	20.43 ± 2.82 ^a^	20.46 ± 2.82 ^a^	20.57 ± 2.74 ^a^
Grip strength (kg)	14.08 ± 8.39	15.43 ± 9.08 *	15.98 ± 9.56 *
Biochemical Parameters			
Albumin (g/dL)	3.51 ± 0.44 ^b^	3.59 ± 0.30 ^ab^	3.66 ± 0.35 ^a^
hs-CRP (mg/dL)	0.86 ± 1.43 ^a^	0.72 ± 1.35 ^a^	0.71 ± 1.02 ^a^
Fasting blood sugar (mg/dL)	95.70 ± 16.38 ^a^	93.57 ± 14.44 ^ab^	91.40 ± 13.12 ^b^
HbA1c (% of Hb)	5.70 ± 0.48 ^a^	5.70 ± 0.55 ^a^	5.70 ± 0.47 ^a^
HOMA-IR index	2.25 ± 3.79 ^a^	2.16 ± 2.26 ^a^	1.97 ± 1.59 ^a^
Triglyceride (mg/dL)	99.63 ± 44.00 ^a^	85.40 ± 33.30 ^b^	84.23 ± 33.82 ^b^
Total Cholesterol (mg/dL)	142.00 ± 34.13 ^a^	139.77 ± 31.98 ^a^	138.57 ± 25.79 ^a^
HDL-c (mg/dL)	39.77 ± 7.56 ^a^	40.17 ± 5.97 ^a^	40.67 ± 7.46 ^a^
LDL-c (mg/dL)	85.30 ± 31.34 ^a^	84.03 ± 29.55 ^a^	81.47 ± 23.86 ^a^

Data are presented as mean ± standard deviation. Different superscript letters in the same group indicate statistically significant differences. * *p* value indicates a significant difference (*p* < 0.05) between the baseline and week 4 or week 8 (paired *t*-test). BMI, body mass index; MAC, mid-arm circumference; MAMC, mid-arm muscle circumference; hs-CRP, high-sensitivity C-reactive protein; HDL, high-density lipoprotein; LDL, low-density lipoprotein.

**Table 5:** BFI-T results.

	Baseline	Week 4	Week 8
Fatigue Severity			
Fatigue right now	5.93 ± 1.96	5.61 ±1.50 *	5.07 ± 1.68 *
Usual fatigue in the last 24 h	5.23 ± 2.46	4.93 ±1.98 *	4.43 ± 1.85 *
The worst fatigue in the last 24 h	6.87 ± 1.78	6.71 ±1.54 *	6.07 ± 1.74 *
Functional Scales			
General activities	4.93 ± 2.30	4.82 ± 1.96 *	4.32 ± 1.72 *
Mood	3.80 ± 2.38	3.54 ± 2.17	3.00 ± 1.76 *
Walking	3.73 ± 2.55	3.57 ± 2.20	3.04 ± 1.82 *
Normal work	6.07 ± 2.12	5.96 ± 1.71 *	5.32 ± 1.85 *
Relations with other people	3.47 ± 2.69	3.43 ± 2.59 *	2.89 ± 2.10 *
Enjoyment of life	5.63 ± 2.37	5.61 ± 1.85 *	4.79 ± 1.62 *
Overall fatigue score	5.07 ± 2.01	4.59 ± 2.02 *	4.04 ± 1.76 *

Data are presented as mean ± standard deviation. * *p* value indicates a significant difference (*p* < 0.05) between the baseline and week 4 or week 8. The overall fatigue score was calculated as the average of fatigue severity and functional scales.

**Table 6:** EROTC QLQC-30.

	Baseline	Week 4	Week 8
Global Health Status/QoL			
Global health status/QoL	44.72 ± 14.60	49.72 ± 12.66 *	51.94 ± 12.12 *
Functional Scales			
Physical functioning	25.78 ± 22.74	38.00 ± 19.11 *	50.44 ± 20.39 *
Role functioning	27.22 ± 28.19	32.78 ± 26.07 *	47.22 ± 30.98 *
Cognitive functioning	56.11 ± 21.66	72.22 ± 24.11 *	76.11 ± 26.87 *
Emotional functioning	60.00 ± 19.38	80.00 ± 23.43 *	81.11 ± 24.07 *
Social functioning	37.78 ± 22.29	37.22 ± 19.42	51.11 ± 23.13 *
Symptom Scales			
Fatigue	65.93 ± 17.25	45.56 ± 20.91 *	34.81 ± 29.29 *
Pain	43.89 ± 19.81	25.00 ± 27.60 *	25.56 ± 27.93 *
Nausea and vomiting	18.33 ± 18.23	16.11 ± 18.30	16.11 ± 18.30
Dyspnoea	28.89 ± 25.87	20.00 ± 25.67 *	16.67 ± 25.89 *
Insomnia	30.00 ± 23.73	15.56 ± 20.96 *	16.67 ± 20.99 *
Appetite loss	27.78 ± 21.59	15.56 ± 16.91 *	16.67 ± 16.95 *
Constipation	32.22 ± 16.34	16.67 ± 19.08 *	13.33 ± 18.77 *
Diarrhea	17.78 ± 16.91	14.44 ± 16.80	15.56 ± 19.04
Financial difficulties	48.89 ± 19.04	58.89 ± 16.80 *	43.33 ± 17.83 *

Data are presented as mean ± standard deviation. * *p* value indicates a significant difference (*p* < 0.05) between the baseline and week 4 or week 8 (paired *t*-test).

## Data Availability

The original contributions presented in this study are included in the article. Further inquiries can be directed to the corresponding author.

## Abbreviations

The following abbreviations are used in this manuscript:

BFI-T, Brief Fatigue Inventory; BMI, body mass index; CRF, cancer-related fatigue; EAA, essential amino acid; EORTC, European Organization for Research and Treatment of Cancer; ESPEN, European Society for Clinical Nutrition and Metabolism; FOHP-ONS, fish oil-enriched high-protein oral nutritional supplement; HDL-C, high-density lipoprotein cholesterol; HOMA-IR, homeostasis model assessment of insulin resistance; hs-CRP, high-sensitivity C-reactive protein; IBW, ideal body weight; LDL-C, low-density lipoprotein cholesterol; MAC, mid-arm circumference; MAMC, mid-arm muscle circumference; MNT, medical nutrition therapy; ONSs, oral nutritional supplements; PDCAAS, protein digestibility-corrected amino acid score; PG-SGA, Scored Patient-Generated Subjective Global Assessment; SPSS, Statistical Package for the Social Sciences; TSF, triceps skinfold thickness.
